# Mandibular Second Premolars with Three Root Canals: A Review and 3 Case Reports

**Published:** 2011-11-15

**Authors:** Zahra Borna, Saeed Rahimi, Shahriar Shahi, Vahid Zand

**Affiliations:** 1. Endodontist, Dental School, Tabriz University of Medical Sciences, Tabriz, Iran.; 2. Department of Endodontics, Dental and Periodontal Research Center, Dental School, Tabriz University of Medical Sciences, Tabriz, Iran.; 3. Department of Endodontics, Dental School, Tabriz University of Medical Sciences, Tabriz, Iran.

**Keywords:** Dental Pulp Cavity/Abnormality, Bicuspids

## Abstract

Before initiating any endodontic therapy, probability of extra canals should be considered. The Incidence of three canals in mandibular second premolar has been reported to be 0.46-0.5%. The present report describes nonsurgical endodontic treatment of three mandibular second premolars with three canals. In these cases, three orifices were located in mesiobuccal, distobuccal and lingual. Mesiobuccal orifices were found after removing dentinal shelves. Even in teeth with extremely complex root canal morphologies, conventional endodontic treatment without surgical intervention can result in adequate healing, as in these cases. Clinicians should be aware of unusual root canal anatomy in mandibular premolars. Very careful examination of the pulpal space, preferably with an optical device is recommended to locate any unusual orifices.

## INTRODUCTION

Successful endodontic treatment requires an understanding of root canal anatomy and morphology. There is wide morphological divergence in the root canal system. Usually clinicians have a thorough understanding of normal anatomy and common variations. Clinicians should be able to identify teeth with different morphologies such as mandibular premolars. According to Green the highest incidence (47%) of accessory foramina was observed in mandibular second premolars [1]. Rahimi et al. also reported high incidence of lateral canals (38.7%) and apical delta (4.38%) in mandibular second premolars [2].

Vertucci et al. reported that the mandibular second premolar had one root canal at the apex in 97.5% and two canals in only 2.5% of the teeth; however, three root canals were scarce [3]. Zillich and Dowson found the incidence of three canals in mandibular second premolars to be 0.4%, which emphasizes the occurrence as being scarce [4]. There seems to be a racial difference for the presence of two or more canals in mandibular premolars. Incidence of mandibular premolars with more than one root canal has been significantly higher in Negroids (32.8%) than in Caucasians (13.7%) [5]. In one study [6], there were mandibular premolars with two canals in 1.6% of Caucasians and 2.6% in Negroids. In spite of the scarce prevalence, the clinician should be aware of these variations, their clinical and radiographic anatomy, and the location of orifices.

The purpose of present case series was to discuss the treatment recommendations for an unusual occurrence of three canals in second mandibular premolars in three patients.

## CASE REPORT

### Case 1:

A 23-year-old male patient with no systemic history was referred to the Department of Endodontics at Tabriz University of Medical Sciences.

**Figure 1:**
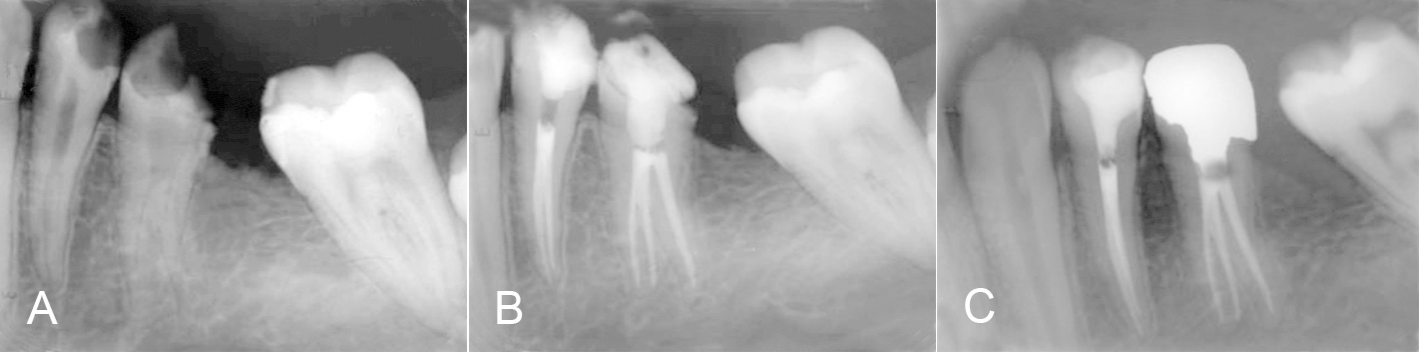
A: Three-canal second premolar. B: Immediate post operative radiograph showing three canals with three portals of exit. C: One-year recall.

The chief complaint of the patient was pain in the lower left back teeth. Clinical examination revealed caries in teeth #20, 21. Vitality tests on both teeth showed painful response to cold, heat and electric pulpal test (EPT) and normal response to percussion. Radiographic examination showed normal periodontium and more than one root canal was suspected in both teeth (Figure 1A).

A pulpal diagnosis of irreversible pulpitis and a periradicular diagnosis of normal were made. Nonsurgical endodontic treatment was planned in both #20 and #21 teeth in one visit. After the administration of the local anesthetic agent (2% lidocaine with 1: 100,000 epinephrine), under rubber dam isolation both #20 and #21 teeth were accessed.

In both teeth two main canal orifices were found. After visualization under surgical operating microscope (OPMI pico Dental Microscope, Zeiss, Oberkochen, Germany) and staining with methylene blue presence of a third canal in #20 was detected.

Working length was established with the use of an apex locator (Root ZX, J. Morita Inc., USA) and confirmed by a radiograph. The canals of teeth #20 were cleaned and shaped with hand K-files (Maillefer Dentsply, Baillaigues, Switzerland) and RaCe NiTi rotary file in a crown down manner up to final canal size of #0.06/30 in lingual canal and #0.06/25 in mesiobuccal and distobuccal canals. The canals were irrigated with 2.5% sodium hypochlorite during instrumentation and 17% EDTA at the end of instrumentation. After final rinse with normal saline, canals were dried and obturated with gutta-percha and AH26 sealer (Dentsply, De Trey, Konstanz, Germany) using the lateral compaction method (Figure 1B).

Both teeth (#20,21) were asymptomatic and had normal periapical condition on radiographs in one-year follow-up (Figure 1C).

### Case 2:

**Figure 2:**
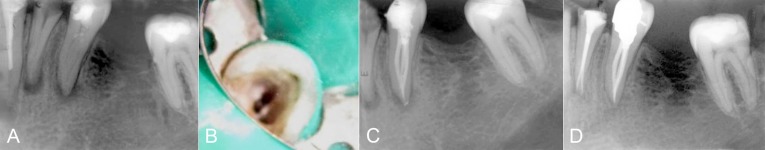
A: Preoperative radiograph of three-canal second mandibular premolar with periapical lesion. B: Access reveals two buccal canals and one lingual canal. C: Post operative radiograph after treatment. D: Six month fallow-up.

A 30-year-old (medically healthy) male patient was referred to the Department of Endodontics. The reason for endodontic treatment was a symptom free necrotic pulp under a leaking composite filling in tooth #20. On the radiograph a bifurcated root could be seen with at least two canals and there was evidence of periapical radiolucency (Figure 2A).

The patient was anesthetized with 2% lidocaine and 1:100,000 epinephrine. After rubber isolation, access to the pulp chamber was made. Two orifices were immediately found on a line connecting buccal cusp and lingual groove. With operating microscope (OPMI pico Dental Microscope, Zeiss, Oberkochen, Germany) at ×10 magnification the dentinal shelves that overlaid orifices were removed with Gates Glidden #3 and #2 with brushing motion on the mesial aspect of the buccally positioned found canal, a third canal was detected, and determined as the mesiobuccal canal (Figure 2B).

After determination of working length with an apex locator (Root ZX) and an additional radiograph with K-file #15 to confirm root canal lengths, chemo-mechanical preparation and obturation of the canals were performed similar to case 1 (Figure 2C).

At six month follow-up session the tooth was symptom-free and periapical lesion was healed (Figure 2D).

### Case 3:

**Figure 3:**
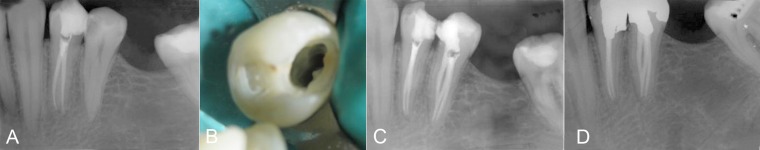
A: Second premolar with three root canals. B: Three orifices were located in the mesial half of the tooth C: Post-operative radiograph after treatment. D: Six month follow-up.

Endodontic non-surgical treatment was performed on tooth #29 in a 25-year-old male patient. No medical history was reported for the patient. The chief compliant was pain in the lowerright back teeth. After performing vitality and percussion tests and radiographic examination, the diagnosis of irreversible pulpitis with normal periapical status was made (Figure 3A).

On preoperative periapical radiograph, presence of two canals was observed with bifurcation on cervical one third of the root. After administration of local anesthesia (lidocaine 2% and 1:100,000 epinephrine) and preparation of the access cavity, two canals were found in buccal and lingual aspects, both located in the mesial half of the tooth. After establishing the working length and chemo-mechanical preparation of the two canals found in a similar manner to the two previous cases, canals were dried and a careful inspection of pulp floor and walls under operating microscope was performed. This revealed a third canal which could be negotiated with a 0.08 K-file (Maillefer) (Figure 3B). Working length was determined by an apex locator and confirmed by radiograph.

The third canal was cleaned and shaped, and obturation of the three canals was accomplished by lateral compaction technique with gutta-percha and AH26 sealer (Figure 3C).

The tooth was symptom-free and had normal periapex on radiograph in six-month follow-up (Figure 3D).

## DISCUSSION

Mandibular second premolar is one of the most difficult teeth for the endodontic treatment [7] because of the variations in internal morphology, extra root canals, apical deltas and lateral canals [8][9].

Straight and angled preoperative radiographs using parallel technique are essential in providing insight into the number of existing root canals [10]. Generally, in mandibular premolars with three canals, the cervical half of the root is wider than usual, with little or no taper [9]. Root canals may not be evident in radiographs and may look unusual. Sudden change in radiographic density and sudden narrowing of root canal space usually indicates an additional canal [11]. Therefore, careful interpretation of the periodontal ligament space and angled views many suggest the presence of an extra root or canal. In the presented cases, unusual root shape was observed in pretreatment radiographs, which recommended the possibility of extra roots and canals.

Using magnifier loupe, fiber optic illuminationfor observation of anatomical land marks in the pulp chamber, sodium hypochlorite bubbling in the extra canals and dyes may be helpful in locating additional canals [12][13]. For better visualization, we used a surgical operating microscope as well as staining with methylene blue, which could penetrate into the orifice to detect developmental grooves, and then according to pulpal floor map, we were able to predict suspected location of canal orifices. In the pulp chamber floor of the mandibular premolars with three canals, many authors have reported one orifice in the lingual side and two in the buccal [9][14][15]. In our reported cases, such a pulpal map was observed.

In the second and the third cases reported, a dentin protuberance of the mesiobuccal wall covered the canal orifice, and root canal entrance was achieved after removing it.

For more reliable working length determination, we use apex locator in combination with radiographs [3]. Two canals in the buccal root in the first case had type IV configuration according to Vertucci classification, while the second and the third cases were type II [16].

We accomplished obturation of all three canals contemporaneously with lateral compaction technique for better results.

Although in vitro and in vivo studies [17][18][19] report low incidence of mandibular second premolars with three canals, each case should be analyzed individually through precise radiographic and clinical examination in order to find all root canals.

## CONCLUSION

Successful and predictable endodontic treatment requires knowledge of normal anatomy and variations. In the case where radiographic images are not helpful to clarify root canal anatomy and aberrations, magnification devices are recommended. Also enhancement of color contrast by means of dye may be helpful to visualize deeply situated orifice and aberrations.

## References

[1] Geen D (1960). Stereomicroscopic study of 700 roots apices of maxillary and mandibular posterior teeth. Oral Surg Oral Med Oral Pathol.

[2] Rahimi S, Shahi S, Yavari HR, Reyhani MF, Ebrahimi ME, Rajabi E (2009). A stereomicroscopy study of root apices of human maxillary central incisors and mandibular second premolars in an Iranian population. J Oral Sci.

[3] Vertucci FJ, Seling A, Gillis R (1974). Root canal morphology of the human maxillary second premolar. Oral Surgery, Oral Medicine, Oral Pathol.

[4] Zillich R, Dowson J (1973). Root canal morphology of mandibular first and second premolars. Oral Surg Oral Med Oral Pathol.

[5] Trope M, Elfenbein L, Tronstad L (1986). Mandibular premolars with more than one root canal in different race groups. J Endod.

[6] Amos ER (1955). Incidence of bifurcated root canals in mandibular bicuspids. J Am Dent Assoc.

[7] Awawdeh LA, Al-Qudah AA (2008). Root form and canal morphology of mandibular premolars in a Jordanian population. Int Endod J.

[8] De Moor RJ, Calberson FL (2005). Root canal treatment in a mandibular second premolar with three root canals. J Endod.

[9] Nallapati S (2005). Three canal mandibular first and second premolars: a treatment approach. J Endod.

[10] Silha RE (1968). Paralleling long cone technic. Dent Radiogr Photogr.

[11] Slowey RR (1979). Root canal anatomy. Road map to successful endodontics. Dent Clin North Am.

[12] Carr GB (1992). Microscopes in endodontics. J Calif Dent Assoc.

[13] Nallapati S, Glassman G (2004). Ophthalmic dyes for root canal location. Endodontic practice.

[14] Chan K, Yew SC, Chao SY (1992). Mandibular premolar with three root canals--two case reports. Int Endod J.

[15] Rödig T, Hülsmann M (2003). Diagnosis and root canal treatment of a mandibular second premolar with three root canals. Int Endod.

[16] Vertucci FJ (1984). Root canal anatomy of the human permanent teeth. Oral Surg Oral Med Oral Pathol.

[17] Al-Fouzan KS (2001). The microscopic diagnosis and treatment of a mandibular second premolar with four canals. Int Endod J.

[18] Macri E, Zmener O (2000). Five canals in a mandibular second premolar. J Endod.

[19] Cleghorn BM, Christie WH, Dong CC (2008). Anomalous mandibular premolars: a mandibular first premolar with three roots and a mandibular second premolar with a C-shaped canal system. Int Endod J.

